# Characterization of sequence variability hotspots in Cranichideae plastomes (Orchidaceae, Orchidoideae)

**DOI:** 10.1371/journal.pone.0227991

**Published:** 2020-01-28

**Authors:** Eric de Camargo Smidt, Michelle Zavala Páez, Leila do Nascimento Vieira, Juan Viruel, Valter Antônio de Baura, Eduardo Balsanelli, Emanuel Maltempi de Souza, Mark W. Chase

**Affiliations:** 1 Departamento de Botânica, Universidade Federal do Paraná, Curitiba, Paraná, Brazil; 2 Royal Botanic Gardens, Kew, Richmond, Surrey, England, United Kingdom; 3 Departamento de Bioquímica, Universidade Federal do Paraná, Núcleo de Fixação Biológica de Nitrogênio, Curitiba, Paraná, Brazil; 4 Department of Environment and Agriculture, Curtin University, Perth, Western Australia, Australia; The National Orchid Conservation Center of China; The Orchid Conservation & Research Center of Shenzhen, CHINA

## Abstract

This study reports complete plastome sequences for six species of Neotropical Cranichideae and focuses on identification of the most variable regions (hotspots) in this group of orchids. These structure of these six plastomes is relatively conserved, exhibiting lengths ranging between 142,599 to 154,562 bp with 36.7% GC on average and exhibiting typical quadripartite arrangement (LSC, SSC and two IRs). Variation detected in the LSC/IR and SSC/IR junctions is explained by the loss of *ndhF* and *ycf1* length variation. For the two genera of epiphytic clade in Spiranthinae, almost whole sets of the *ndh*-gene family were missing. Eight mutation hotspots were identified based on nucleotide diversity, sequence variability and parsimony-informative sites. Three of them (*rps16-trnQ*, *trnT-trnL*, *rpl32-trnL*) seem to be universal hotspots in the family, and the other five (*trnG-trnR*, *trnR-atpA*, *trnP-psaJ*, *rpl32-infA*, and *rps15-ycf1*) are described for the first time as orchid molecular hotspots. These regions have much more variation than all those used previously in phylogenetics of the group and offer useful plastid markers for phylogenetic, barcoding and population genetic studies. The use of whole plastomes or exclusive no-gap matrices also positioned with high support the holomycotrophic *Rhizanthella* among Orchidoideae plastomes in model-based analyses, showing the utility of plastomes for phylogenetic placement of this unusual genus.

## Introduction

Orchidaceae are one of the largest families of flowering plants, including five subfamilies, 736 genera and more than 30,000 species [1, 2]. One of the five subfamilies is Orchidoideae, divided into four tribes, including Cranichideae. As currently recognized, this cosmopolitan subtribe has eight subtribes and includes 96 genera with ca. 1,400 spp [1, 3]. They are characterized by their fleshy, but not tuberose, fasciculate roots, clustered or distributed along rhizome [3].

Many studies have dealt with phylogenetic issues in the tribe, some in a more comprehensive way focusing on revised limits of the subtribes or involving circumscription or new positions of genera [4–18], among others. In these studies, largely based on plastid markers, the nuclear ribosomal internal transcribed spacers (nrITS) have been the exclusive source of nuclear information in the tribe (exception being [18], who used *Xdh* in Goodyerinae). Most of these studies used only a few plastid regions, usually *rbcL* and *matK* exons, the intron/spacer of *trnL-F* and less frequently the *atpB* and *psaB* exons and the *rpoB-trnC* spacer. Use of these regions was purely based on their previous use in other studies without prior analysis of their variability. Moreover, the low resolution and support in several clades in these studies necessitates identification of new and more variable regions for obtaining well-supported results.

In recent years, high-throughput sequencing technologies have provided an opportunity for study of genomic evolution and interspecific relationships of organisms based on large-scale genomic resources, such as complete plastome sequences [19, 20]. Due to their abundance in the cells, generally maternal inheritance and conserved structural characteristics, plastomes can provide information for studying species divergence and interspecific relationships of plants [21]. In most angiosperms, plastomes have a quadripartite structure: two inverted regions (IRs) intercalated between large single-copy (LSC) and small single-copy (SSC) regions [21, 22]. The plastome is circular and usually ranges from 107 to 218 kbp in length with about 120 genes [20]. In the orchid family, the length of the plastome is usually within this range [23, 24], except for holomycotrophic species in which some plastomes have shrunk to 19 kbp [25, 26].

In this study, we aim to identify the most variable plastid regions in this orchid tribe for use in future phylogenetics and population genetics studies of Atlantic Rainforest endemics. To do that, complete plastomes of six Cranichidinae species native to the Neotropics were first assembled and annotated. We analysed the differences in genome size, content, and structure, characterizing sequence divergence in hotspot regions. We also included six previously published Paleotropical Cranichidinae plastomes. In addition, we evaluated the phylogenetic relationships in Orchidoideae based on eighteen plastome datasets to contribute to an understanding the position of these genera with the phylogenomic data available so far. We focus in particular on the Australian underground orchid, holomycotrophic *Rhizanthella gardneri* R.S.Rogers, which has been the subject of phylogenetic controversy due to having one of the most highly reduced plastomes of any orchid sequenced to date, with only 59,910 bp and 33 retained genes [3, 27, 28].

## Material and methods

### Taxon sampling, DNA extraction and sequencing

Fresh leaf material of *Aspidogyne longicornu* (Cogn.) Garay (Goodyerinae) *Prescottia stachyodes* (Sw.) Lindl. (Cranichidinae), *Cyclopogon argyrifolius* (Barb.Rodr.) Barb.Rodr., *Sauroglossum elatum* Lindl., *Eurystyles cotyledon* Wawra, *Lankesterella ceracifolia* (Barb.Rodr.) Mansf. (all Spiranthinae) was collected from southeastern Brazil in the Atlantic Rainforest (Table 1).

**Table 1:** Vouchers and general features of the complete plastomes of Cranichideae species analyzed in this study.

Plastome	Voucher	Total cpDNA Size (bp)	Length of LSC region (bp)	Length of IR regions (bp)	Length of SSC region (bp)	% GC	GenBank accession
*Aspidogyne longicornu*	Engels, M.E. 1504 (UPCB)	153,436	82,437	26,492	18,015	36.9	MN597437
*Cyclopogon argyrifolius*	Carvalho, B.M. 153 (RB)	154,495	83,704	26,506	17,779	36.9	MN597436
*Eurystyles cotyledon*	Imig, D.C. 366 (UPCB)	139,811	79,842	24,396	11,177	36.5	MN597435
*Lankesterella ceracifolia*	Imig, D.C. 358 (UPCB)	142,599	78,992	26,892	9,823	36.3	MN597434
*Prescottia stachyoides*	Smidt, E.C. 1080 (UPCB)	154,044	83,373	26,493	17,685	36.7	MN597433
*Sauroglossum elatum*	Imig, D.C. 502 (UPCB)	154,562	83,822	26,503	17,734	37.0	MN597432

Plastid enrichment was performed according to the protocol developed by [29], adapted for a small amount of tissue [30]. Plastid-enriched DNA was extracted according to [31], scaled to 2 mL, and purified with DNA Clean and Concentrator kit (Zymo Research, Orange, CA). Approximately 1 ng of DNA was used for library preparation using the Nextera XT DNA Sample Prep Kit (Illumina Inc., San Diego, CA), following manufacturer’s instructions. Sequencing was performed on an Illumina MiSeq (Illumina Inc., San Diego, CA). The number of paired reads (2 × 250 bp) per sample varied from 501,686 to 1,637,814 and were imported as FASTQ format in CLC Genomics Workbench v.11.0 (http://www.qiagenbioinformatics.com). Reads were trimmed using a 0.05 quality threshold, resulting in 498,811 to 1,534,494 reads of 167 bp length on average (S1 Table). These reads were assembled into contigs in CLC Genomics Workbench (v. 8 and 12) and DNASTAR Lasergene v. 14. The correct size and positioning of the IRs were determined using REPuter [32], and the IR boundaries were analyzed and visually compared in Geneious Prime 2019.1.1 (https://www.geneious.com). Annotations were manually checked: protein-coding regions were validated with ExPASy [33] and tRNA with tRNAscan-SE 2.0 [34]. The graphic representation of the plastomes was then produced using OGDRAW [35].

Plastome structure, gene content, and general characteristics of the plastid genomes sequenced were compared with three published plastomes of the same subtribe from the Paleotropics available on the NCBI website: *Anoectochilus emeiensis* K.Y.Lang (NC033895), *Goodyera fumata* Thwaites (NC026773), and *Goodyera schlechtendaliana* Rchb.f. (NC029364). Sequences were aligned using the progressive Mauve algorithm [36] in Geneious Prime to verify structural differences between plastomes.

Comparison of the number of protein, tRNA and rRNA coding regions was performed by plastome alignment with MAFFT 7 software with the automatic algorithm plugin, 200PAM/k = 2 [37] in Geneious Prime. In order to examine IR junctions, sequence and content of these regions were plotted using the online IRscope tool [38]. DnaSP v5.10.1. [39] was employed to assess sequence variability (SV) among each protein-coding gene (CDS), intron, and intergenic spacer (IGS) of more than 150 bp flanked by the same region. The number of mutations, insertion/deletion (indel) events, and conserved sites were counted for each sequence. SV was calculated according to the method of [40]: SV = (number of nucleotide mutations + the number of indel events)/(the number of conserved sites + the number of nucleotide mutations + the number of indel events) × 100%. The number of potentially parsimony informative sites (PIS) and nucleotide diversity (Pi) were obtained as calculated in DnaSP. The nrITS sequences of the same species were obtained from GenBank to compare the levels of variation in various plastid regions with this most widely used nuclear marker in Orchidaceae (except for the use of *Anoectochilus roxburghii* (Wall.) Lindl.—GQ32877 instead of *A*. *emeiensis* due the lack of an available nrITS).

### Phylogenetic analyses

For phylogenomic analyses, we used as an outgroup the plastome of *Cypripedium japonicum* Thunb. (NC027227), subfamily Cypridedioideae. We also included the available plastomes from subfamily Orchidoideae, tribe Orchideae, subtribe Orchidinae: *Dactylorhiza majalis* (Rchb.) P.F.Hunt & Summerh. (MK984209), *Habenaria pantlingiana* Kraenzl. (NC026775), *Ophrys sphegodes* Mill. (AP018717), *Platanthera chlorantha* (Custer) Rchb. (MK937914), *P*. *japonica* (Thunb.) Lindl. (NC037440); tribe Diurideae, subtribe Rhizanthellinae: *Rhizanthella gardneri* R.S.Rogers (GQ413967); and tribe Cranichideae, Subtribe Goodyerinae: *Anoectochilus emeiensis* K.Y.Lang (NC033895), *Goodyera fumata* Thwaites (NC026773), *Goodyera procera* (Ker Gawl.) Hook. (NC029363), *Goodyera schlechtendaliana* Rchb. f. (NC029364), *Goodyera velutina* Maximowicz ex Regel (NC029365), and *Ludisia discolor* (Ker Gawl.) Blume (NC030540). Multiple sequence alignments were performed using MAFFT 7 [37] with settings described above, visually inspecting and manually adjusting them with Geneious Prime. Indels were treated as missing data. Phylogenetic analyses were performed using maximum likelihood (ML), maximum parsimony (MP), and Bayesian inference (BI) in order to explore the results with different assumptions. Due to the loss of many plastid genes in *R*. *gardneri*, we repeated the phylogenetic analyses with only non-gap positions to reduce the effect of missing data on the phylogenetic placement of this species. ML trees were calculated using IQ-tree 1.6.11 [41] with the concatenated unpartitioned scheme and tree search with 1,000 bootstrap replicates in a single run [42, 43]. The best-fit models of substitution inferred in the “supergene” analyses by AIC were GTR+F+R5. For the support, we used the ultrafast bootstrap approximation (UFBoot) [44], with the strategy (-bnni) to reduce the risk of overestimating support. Also, we calculated the SH-like approximate likelihood ratio test (-alrt 1000) [45] as an alternative method of estimating support. We accepted only SH-aLRT ≥ 80% and UFboot ≥ 95% as a good support. MP analyses were performed with Fitch parsimony [46] using the software PAUP 4.0b10a [47]. Analyses included 1,000 random taxon-addition replicates, holding 10 trees per replicate, TBR swapping algorithm, followed by a second search to explore all topologies from the previous search, limited to 10,000 trees. Support was estimated with 1,000 bootstrap replicates [48], simple addition, TBR algorithm and holding 10 trees per replicate. BI was performed using MrBayes 3.2.6 [49] with GTR+I+G model. The analyses started from random trees and employed Markov chain Monte Carlo (MCMC) runs, over ten million generations, sampling trees every 1000 generations. We discarded 25% of the initial generations as the burn-in, after visual inspection for stabilization of tree log-likelihood, as measured by the standard deviation(s) and PSRF (potential scale reduction factor) values [50] and ESS >300 in the Tracer 1.7 [51]. The remaining trees were used to produce the BI tree with posterior probabilities. All trees were edited with FigTree 1.4.0 [52].

## Results

### Plastome description and hotspot characterization

**Fig 1:**
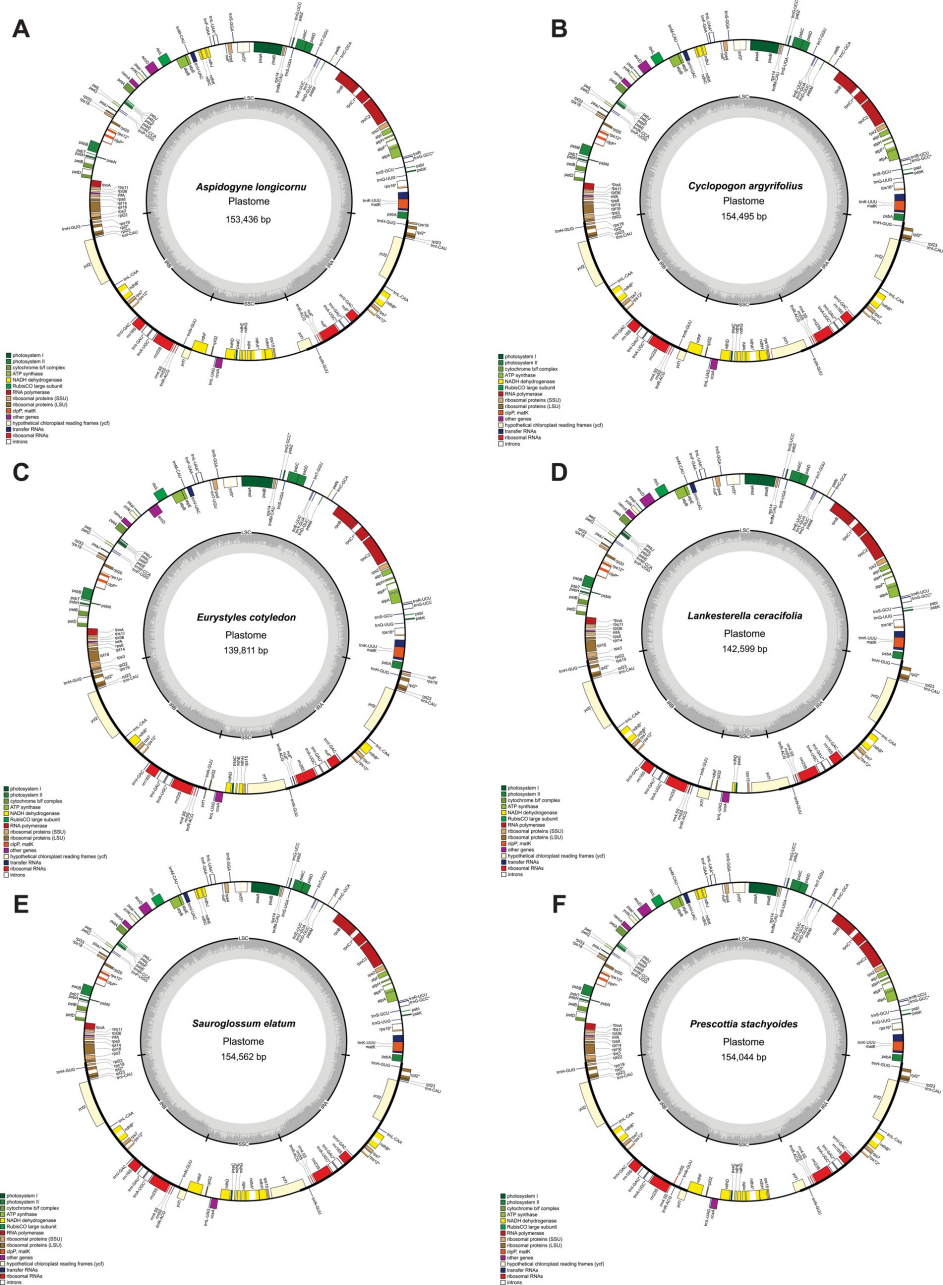
Cranichideae plastomes circular map. A, *Aspidogyne longicornu*, B, *Cyclopogon argyrifolius*, C, *Eurystyles cotyledon*, D, *Lankesterella ceracifolia*, E, *Prescottia stachyoides*, F, *Sauroglossum elatum*. The genes represented inside the circle are transcribed clockwise and those outside are transcribed counter-clockwise. The dark gray rim inside the figure corresponds to the distribution of GC content across the plastome.

The six newly sequenced plastomes exhibited lengths ranging from 139,811 to 154,562 bp, with 36.7% average GC content and the typical quadripartite structure (Table 1, Fig 1).

**Fig 2:**
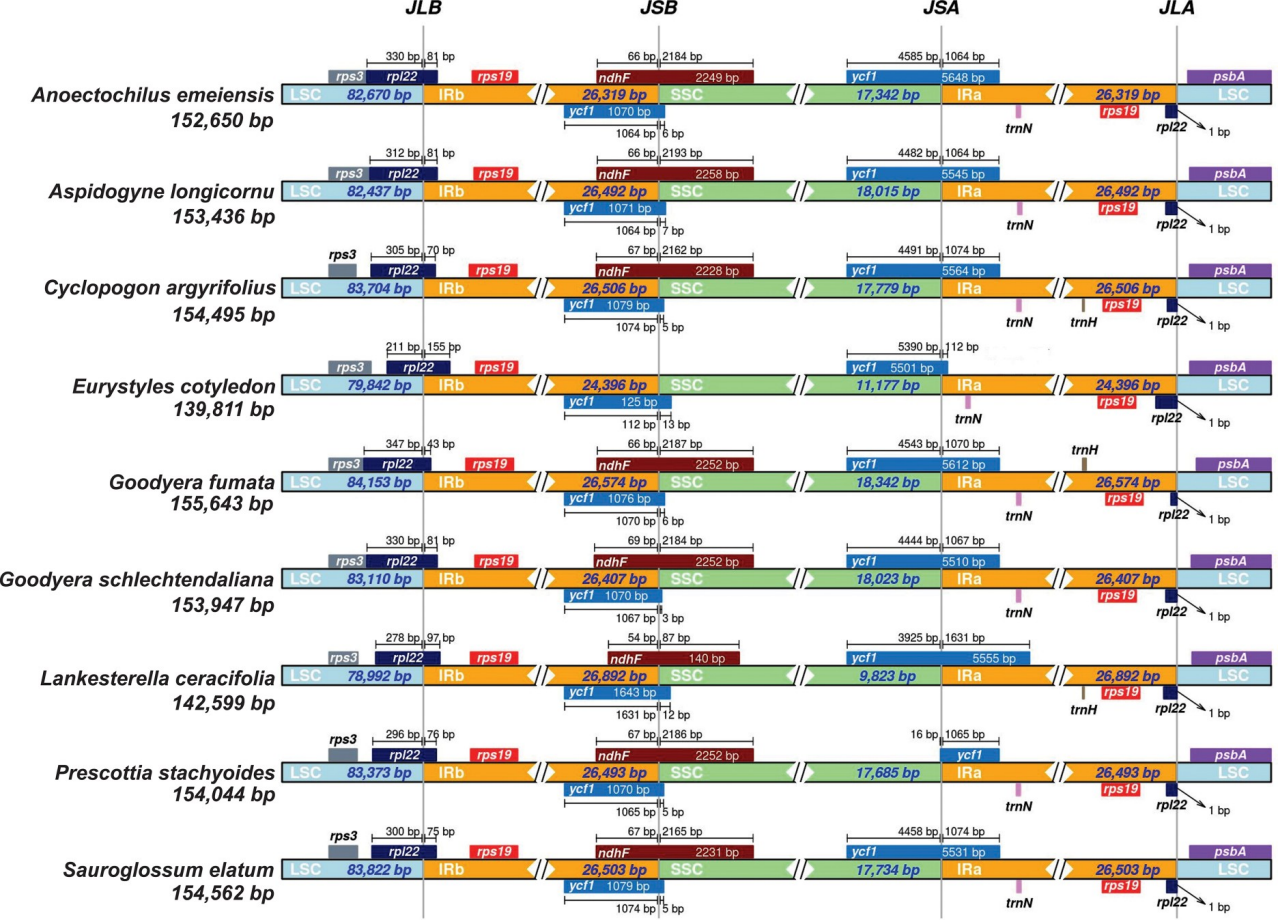
Comparison of the boundaries of LSC, SSC, and IR regions of the complete plastomes of Cranichideae. JLB (IRb /LSC), JSB (IRb/SSC), JSA (SSC/IRa) and JLA (IRa/LSC) denote the junctions between each corresponding region in the genome.

The Mauve alignment indicated that there are no major structural differences between the analyzed plastomes (S1 Fig). Variation in the length of some regions (S2 Table) and differences in the positioning of some genes in IR boundaries (Fig 2) was identified.

The list of plastid genes identified is presented in Table 2, and statistics for each CDS, intron, and intergenic spacer are in S2 Table.

**Table 2:** List of genes identified in the plastomes of *Aspidogyne longicornu*, *Cyclopogon argyrifolius*, *Eurystyles cotyledon*, *Lankesterella ceracifolia*, *Prescottia stachyoides* and *Sauroglossum elatum*.

Group of gene	Gene name
Gene expression machinery	
Ribosomal RNA genes	*rrn4*.*5*^b^; *rrn5*^b^; *rrn16*^b^; *rrn23*^b^
Transfer RNA genes	*trnA–*UGC^a^^b^; *trnC–*GCA; *trnD–*GUC; *trnE–*UUC; *trnF–*GAA; *trnfM–*CAU; *trnG–*GCC^b^; *trnG–*UCC; *trnH–*GUG^b^; *trnI–*CAU^b^; *trnI–*GAU^a^^b^; *trnK–*UUU; *trnL–*CAA^b^; *trnL–*UAA^a^; *trnL–*UAG; *trnM–*CAU; *trnN–*GUU^b^; *trnP–*UGG; *trnQ–*UUG; *trnR–*ACG^b^; *trnR–*UCU; *trnS–*GCU; *trnS–*GGA; *trnS–*UGA; *trnT–*GGU; *trnT–*UGU; *trnV–*GAC^b^; *trnV–*UAC^a^; *trnW–*CCA; *trnY–*GUA
Small subunit of ribosome	*rps2*; *rps3*; *rps4*; *rps7*^b^; *rps8*; *rps11; rps12*^a^^b^*; rps14*; *rps15*; *rps16*^a^; *rps18*; *rps19*^b^
Large subunit of ribosome	*rpl2*^a^^b^; *rpl14*; *rpl16*^a^; *rpl20*; *rpl22* ^b^; *rpl23*^b^*; rpl32; rpl33; rpl36*
DNA-dependent RNA polymerase	*rpoA*; *rpo*^b^; *rpoC1*^a^; *rpoC2*
Translational initiation factor	*infA*
Maturase	*matK*
Genes for photosynthesis	
Subunits of photosystem I (PSI)	*psaA*; *psaB*; *psaC*; *psaI*; *psaJ*; *ycf3*^a^; *ycf4*
Subunits of photosystem II (PSII)	*psbA*; *psbB*; *psbC*; *psbD*; *psbE*; *psbF*; *psbH*; *psbI*; *psbJ*; *psbK*; *psbL*; *psbM*; *psbN*; *psbT*; *psbZ*
Subunits of cytochrome *b*_*6*_*f*	*petA*; *petB*^a^; *petD*^a^; *petG*; *petL*; *petN*
Subunits of ATP synthase	*atpA*; *atpB*; *atpE*; *atpF*^*a*^; *atpH*; *atpI*
Subunits of NADH dehydrogenase	*ndhA*^a^^c^^d^; *ndhB*^a^^b^^c^; *ndhC*^c^^d^; *ndhD*^c^^d^; *ndhE*^d^; *ndhF*^c^^d^; *ndhG*^c^^d^; *ndhH*^c^^d^; *ndhI*^c^^d^; *ndhJ*^c^^d^; *ndhK*^c^^d^
Large subunit of Rubisco	*rbcL*
Other functions	
Envelope membrane protein	*cemA*
Subunit of acetyl-CoA carboxylase	*accD*
C-type cytochrome synthesis	*ccsA*
Subunit of protease Clp	*clpP*^a^
Component of TIC complex	*ycf1*^b^
Unknown function	*ycf2*^b^

^a^ Genes containing introns

^b^ Duplicated genes

^c^ Deleted in *Eurystyles*

^d^ Deleted in *Lankesterella*

A few length differences in one copy of the *rpl22* gene were detected in the LSC/IRb junction. The *ndhF* region was absent in *Eurystyles cotyledon*, and the length of *ycf1* was shorter than in the other studied species in the IRb/SSC junction. In the SSC junction with IRa, there are differences in the length of the second copy of the *ycf1* and a few differences in the length of *rpl22*.

All genes are present in all species, except for some subunits of NAD(P)H-quinone oxidoreductase, which are missing in *Lankesterella ceracifolia* (*ndhA*, *ndhC*, *ndhE*, *ndhG*, *ndhH*, *ndhI*, *ndhJ*, *nhdK*) and *Eurystyles cotyledon* (*ndhA*, *ndhC*, *nhdF*, *ndhG*, *ndhI*, *ndhJ*, *nhdK*).

**Fig 3:**
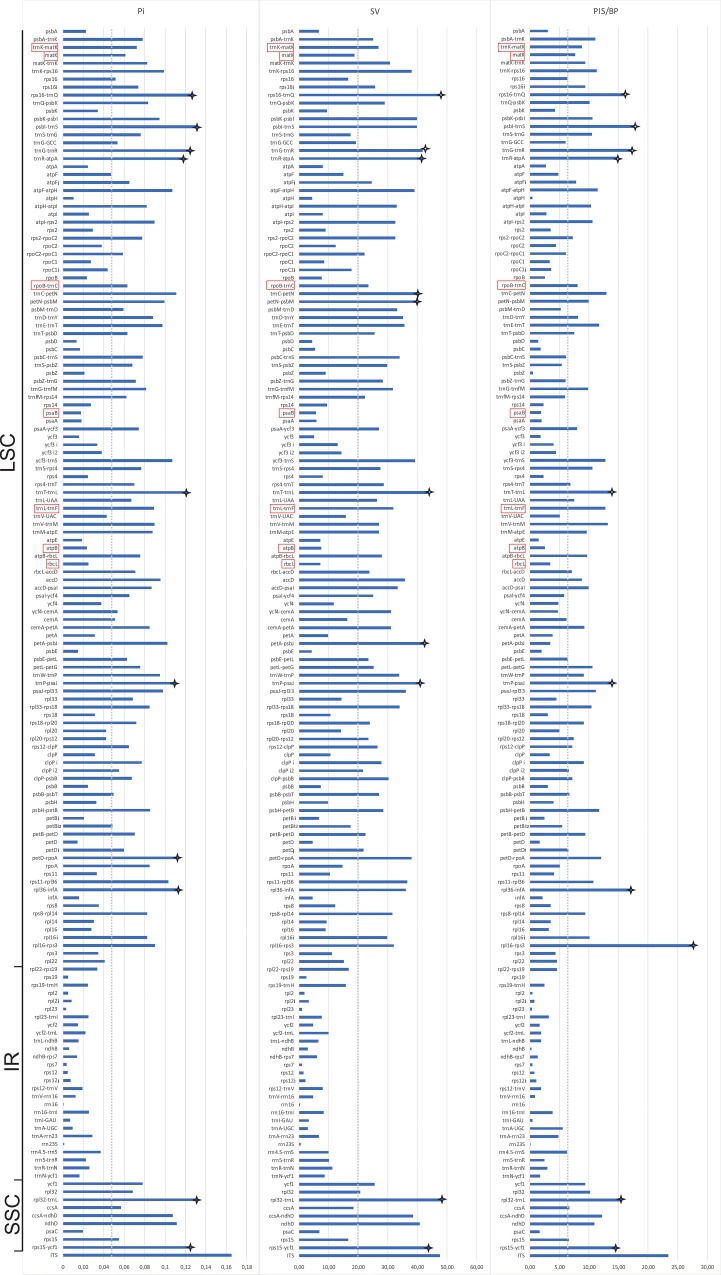
Expected number of genetic differences within each protein-coding sequence (CDS), intron, and intergenic spacer (IGS) of Cranichideae plastomes. Gene order is preserved beginning at the top with the large single-copy (LSC), intersection of the inverted repeat (IR), and the small single-copy (SSC) region at the bottom, ending with *rps15-ycf1*. For comparison, nrITS is placed at the bottom. The vertical dotted lines highlight the average value across regions (Pi = 0.056, SV = 20.14 and PIS/bp = 6.55), and the stars highlight the ten regions that on average contain the greatest amount of nucleotide diversity (Pi), highest sequence variability (SV), and greatest number of potentially informative characters (PIS/bp). Regions in red were used in previous phylogenetic studies of the group.

For all regions longer than 150 bp, the statistics for Pi, SV and PIS/bp are relatively congruent. For the ten most variable regions in each statistic, all are intergenic spacers and eight most variable regions are the same for the three parameters (*rps16-trnQ*, *trnG-trnR*, *trnR-atpA*, *trnT-trnL*, *trnP-psaJ*, *rpl32-infA*, *rps15-ycf1* and *rpl32-trnL*). No individual region has more nucleotide diversity (Pi) than nrITS, but *rpl32-trnL* and *rpS16-trnQ* contain more sequence variability (SV) and *rpL16-rpS6* contains more parsimony-informative sites (Fig 3, S2 Table).

### Phylogenetic relationships

**Fig 4:**
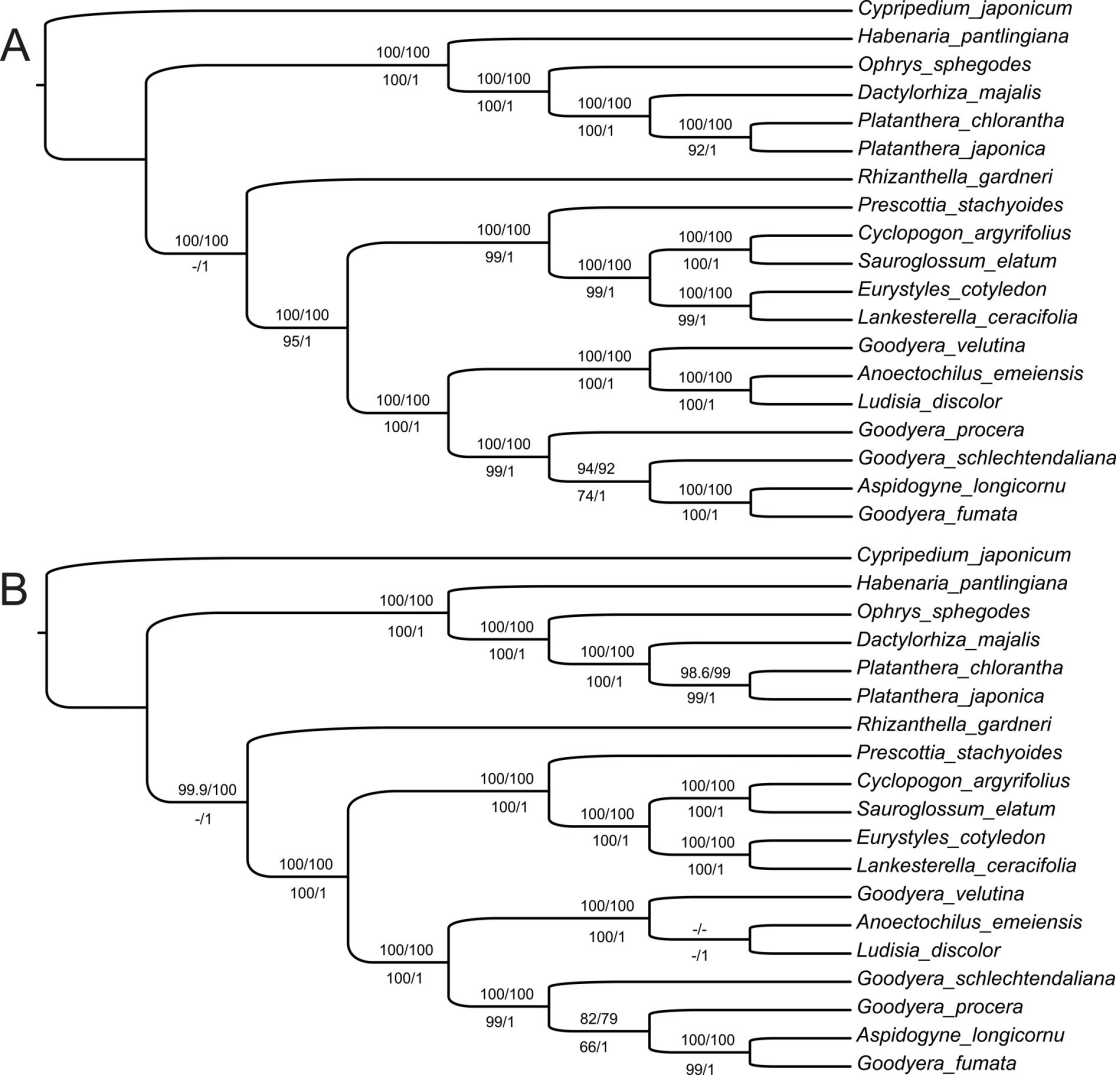
Phylogenetic relationships inferred with ML, MP, and BI. A. Full-length plastome sequences of representative Orchidoideae. B. With only non-gap sites of the plastomes. Node support is derived from IQ-tree bootstrap, and SH-like approximate likelihood ratio test above the branches and maximum parsimony bootstraps and Bayesian posterior probabilities bellow the branches. *Cypripedium japonicum* was specified as the outgroup.

The aligned dataset consisted of 195,841 characters, of which 153,336 (78%) are monomorphic, and 16,086 (8.2%) characters were potentially parsimony-informative. MP recovers only one tree (L = 64,472 steps) with a consistency index of 0.78 and retention index of 0.72. All phylogenetic analyses reconstructed the same topology with strong support for monophyly of tribes Cranichideae and Orchideae (Fig 4). In Cranichideae, Cranichidinae are monophyletic with strong support, *Prescottia stachyoides* is sister of Spiranthinae and both sister to Goodyerinae. In Spiranthinae, *Eurystyles cotyledon* plus *Lankesterella ceracifolia* are sister to *Cyclopogon argyrifolius* plus *Sauroglossum elatum*. In subtribe Goodyerinae, *Goodyera* is biphyletic, and *Aspidogyne* is sister to *G*. *fumata*. All these relationships receive strong support. *Rhizanthella* is sister to Orchideae with high support in the BI and ML trees, but with MP this species is sister to both Cranichideae and Orchideae with low support (not shown).

The matrix excluding gaps consisted of 51,193 characters, of which 36,614 (73%) are constant, and 4,090 (8,1%) characters were potentially parsimony-informative. MP recovered one tree (L = 19,860 steps) with a consistency index of 0.79 and retention index of 0.69. All analyses reconstructed the same topology for complete matrix, except for lack of support for a clade of Goodyerinae that contains *Ludisia discolor*, *Anoectochilus emeiensis* and *Goodyera* (Fig 4). *Rhizanthella* is sister to Orchideae with high support in BI and ML analyses, and with MP this species is again sister to Cranichideae and Orchideae with low support (not shown).

## Discussion

### Plastome description and hotspot characterization

This study sequenced for the first time six whole plastomes for six orchid species in subtribe Cranichideae. The genomic structure and gene content are conserved in general. Plastome length in *Eurystyles cotyledon* and *Lankesterella ceracifolia* were shorter than those of the other species, due to loss of most *ndh* genes encoding subunits of the NAD(P)H-quinone oxidoreductase proteins. The *ndh* gene complex comprises 11 genes that act in electron transport in photosystem I [53]. However, in Orchidaceae plastomes, there is a great variation in retention/deletion of the *ndh* genes among different clades, suggesting independent losses [24, 54–57]. Because they are important genes for photosynthesis, there may be functional copies in the nuclear or mitochondrial genomes [21, 26, 55, 58, 59], but it has also been suggested that the *ndh* genes are dispensable [60]. In several photosynthetic groups that are neither mycoheterotrophic nor parasitic (summarized in [61]), *ndh* genes have been lost and often are the first plastid genes to be lost [26, 62]. *Eurystyles* and *Lankesterella* are the only genera of the epiphytic clade in Spiranthinae (*sensu* [17]), and their *ndh* gene loss should probably be considered an apomorphic character in the relation to the other two Spiranthinae plastomes sequenced. [54, 55] suggested that there is a clear relationship between the loss of *ndh* genes and instability in IR/SSC boundaries, and [24] also reported length variation in *ycf1*. In our study, both conditions are related to differences in these boundaries (Fig 2).

Niu et a. [63] reported mutational hotspots as genus-specific in orchid plastomes and proposed three markers for Epidendroideae (*trnK-rps16*, *trnS-trnG*, and *rps16-trnQ*) and two for Cypripedioideae (*clpP-psbB* and *rps16-trnQ*). In a study of plastome variation in *Dendrobium*, [64] proposed the combination of five markers (*trnT-trnL*, *rpl32-trnL*, *clpP-psbB*, *trnL intron*, and *rps16-trnQ*) for phylogenetic and DNA barcoding studies of the genus. Here, we suggest eight hotspot markers for Cranichideae. Due to previously being regarded in other studies as hot spots, three of them (*rps16-trnQ*, *trnT-trnL*, *rpl32-trnL*) seem to be universal in the family, but the other five (*trnG-trnR*, *trnR-atpA*, *trnP-psaJ*, *rpl32-infA*, and *rps15-ycf1*) are identified for the first time.

### Phylogenetic relationships

In all analyses, the currently recognized taxa (tribes and subtribes) with more than one plastome included in the analysis were recovered with high support and, although with limited taxon sampling, our results agreed with the classification proposed in [1]. In Cranichidinae, as expected, *Prescottia* is sister to the Spiranthinae plastomes sampled, and in Spiranthinae, the epiphytic clade (*Eurystyles* plus *Lankesterella*) is also recovered with high support as sister to *Cyclopogon* plus *Sauroglossum*, in agreement with the results of [17]. By contrast, for the Goodyerinae, although the subtribe is recovered with high support, *Goodyera* is biphyletic. Chen et al. (2019) demonstrated the non-monophyly of *Goodyera* as currently circumscribed using two nuclear and five plastid markers. Our results place *Goodyera fumata* sister to *Aspidogyne*, which corresponds to the *Microchilus* subclade *sensu* [18].

Many non-photosynthetic taxa (both mycotrophic and parasitic) have a history of being phylogenetically difficult to place because many of the plastid regions utilized in early phylogenies are absent or degraded, and there have been such problems as well with holomycotrophic orchids [25, 26, 65, 66]. The position of holomycotrophic *Rhizanthella* in our analyses agrees with the classification scheme of [1], sister to Cranichideae with high ML and BI support and low support with MP, which placed *Rhizanthella* as a sister to both tribes. The full plastome analysis shows better support than the use only non-gaped sites. In addition, absence of some regions in the plastomes of non-photosynthetic taxa species and rate heterogeneity could lead to phylogenetic artifacts [65].

## Conclusions

In this study, six plastid genomes of Cranichideae species were sequenced and annotated. They are relatively conserved, but for the epiphytic clade of Spiranthinae almost the whole set of the *ndh*-gene complex was truncated or pseudogenized. Eight mutation hotspot regions were identified in these plastid genomes and have much more variation than any those used for the group before, giving them potential as markers for phylogenetic, barcoding and population genetic studies. Although different criteria and methods could affect hotspot identification, our findings in this study compared with previous studies in other orchid groups suggest many plastid hotspots in are clade-specific, whereas other hotspots are shared by all orchid genera. The use of complete plastomes supported placement of holomycotrophic *Rhizanthella* as sister to Cranichideae, demonstrating the utility of plastomes in orchid phylogenetics.

## Supporting information

S1 Fig Mauve alignment with the progressive Mauve algorithm of the plastomes. Monochromatic alignment indicates that there is no structural variation among the plastomes.
(TIFF)

S1 Table General features of reads in sequencing of plastomes for Cranichideae. (DOCX)

S2 Table Statistics of the expected number of genetic differences within each protein-coding gene (CDS), introns, and intergenic spacer (IGS) of Cranichideae plastomes. For comparison, nrITS is placed at bottom. The columns indicate: A, position in the plastome, B, Name of the region, C, Region of the plastome (SSC, IR, LSC), D, Type of the region (CDS, IGS, INTRON), E, Nucleotide diversity (Pi), F, Number of conserved sites (CS), G, Number of Mutations (NS), H, Indels Events (ID), I, number of inversions (IV), J, Sequence variability (SV), K, Number of parsimony informative sites (PIS), L, PIS/base pairs, M, Total sequence size (L), N, L less gaps sites.
(XLSX)
