# Supplementary material for: Characterization of sequence variability hotspots in Cranichideae plastomes (Orchidaceae, Orchidoideae)
Source: PLoS One. 2020 Jan 28;15(1):e0227991. doi: 10.1371/journal.pone.0227991 (PMC6986716; doi:10.1371/journal.pone.0227991)
Supplement: S1 Table — (DOCX) [file pone.0227991.s002.docx]

**S1 Table. General features of reads in the sequencing of the complete plastomes of Cranichideae.**

| **Plastome** | **Total number**  **of reads** | **Median**  **Length** | **Remaining**  **reads** | **Reads length**  **after trimming** |
| --- | --- | --- | --- | --- |
| *Aspidogyne longicornu* | 776,122 | 201.3 | 769,395 | 201.6 |
| *Cyclopogon argyrifolius* | 501,686 | 206.8 | 498,811 | 207.4 |
| *Eurystyles cotyledon* | 1,285,132 | 121.1 | 1,172,477 | 127.4 |
| *Lankesterella ceracifolia* | 1,637,814 | 149.7 | 1,534,494 | 156.0 |
| *Prescottia stachyodes* | 1,145,140 | 174.2 | 1,106,984 | 177.9 |
| *Sauroglossum elatum* | 1,429,466 | 179.7 | 1,402,338 | 181.7 |
